# Numerical Characterization of Optical Transmittance Through Planar Multilayer Dielectric Stacks on CMOS Photodiodes

**DOI:** 10.3390/s26206591

**Published:** 2026-10-17

**Authors:** João Fernandes, Gabriel M. Ferreira, João P. Carmo, Graça Minas, Susana O. Catarino

**Affiliations:** 1 Center for Microelectromechanical Systems (CMEMS), School of Engineering, Campus de Azurém, University of Minho, 4800-058 Guimarães, Portugal; pg55955@alunos.uminho.pt (J.F.); id10786@alunos.uminho.pt (G.M.F.); scatarino@dei.uminho.pt (S.O.C.); 2 International Iberian Nanotechnology Laboratory (INL), 4715-330 Braga, Portugal; 3 Group of Metamaterials Microwaves and Optics (GMeta), Department of Electrical and Computer Engineering (SEL), University of São Paulo, Av. Trabalhador S.-Carlense, Nr. 400, São Carlos CEP 13566-590, SP, Brazil; jcarmo@sc.usp.br; 4 LABBELS—Associate Laboratory, 4800-122 Braga, Portugal

**Keywords:** CMOS photodiode, multilayer dielectric stack, optical transmission, thin-film interference, finite element method, ray tracing, transfer-matrix method

## Abstract

Accurately predicting how incident light propagates through complex dielectric layers is essential for optimizing the design and performance of integrated CMOS (Complementary Metal Oxide Semiconductor) silicon photodiodes without relying on additional costly fabrication processes. To address this need, this work presents a comparative analysis between three numerical approaches for modelling the optical transmission through planar multilayer dielectric stacks, representative of TSMC (Taiwan Semiconductor Manufacturing Company) 65 nm and TSMC 180 nm CMOS technologies. Wave Optics and Ray Optics, implemented in COMSOL Multiphysics, together with the transfer-matrix method implemented in TFCalc, were evaluated by comparing spectral transmittance over the 400 to 1000 nm wavelength range, sensitivity to fabrication process-induced thickness variations, angular responses from 0° to 80°, and computational efficiency. The three methods exhibited excellent agreement, accurately reproducing the interference behavior of both dielectric stacks. The TSMC 65 nm technology showed a more complex optical response, whereas the TSMC 180 nm stack presented generally higher transmittance, above 31% throughout the evaluated spectral range. Process tolerances primarily shifted the spectral position of the interference peaks by approximately 90 nm and 80 nm, for TSMC 65 nm and 180 nm technologies respectively, without significantly altering the overall spectral behavior, while angular averaging from 0° to 60° smoothed the transmittance oscillations expected under practical LED (Light-Emitting Diode) illumination. TFCalc achieved near full-wave accuracy while reducing the computational time from nearly four hours to approximately one second, demonstrating the best accuracy–efficiency trade-off for planar multilayer dielectric stack analysis in CMOS photodiode design.

## 1. Introduction

Complementary Metal Oxide Semiconductor (CMOS) silicon photodiodes have become fundamental components in integrated optoelectronic systems due to their low fabrication cost, compatibility with standard CMOS processes, and straightforward integration with on-chip electronic circuits [1,2]. Their widespread use in optical sensing, spectroscopy, imaging, optical communications and biomedical lab-on-a-chip platforms has motivated continuous research to improve their spectral response and broadband operation [3,4], while enabling accurate prediction of device performance during the design stage, reducing the need for costly fabrication iterations [1,5,6,7,8,9].

An intuitive representation of a CMOS photodiode suggests that incident light propagates directly into the silicon active region, where absorbed photons generate electron-hole pairs that contribute to the photocurrent [1,7]. In practice, however, this simplified representation does not accurately describe standard CMOS technologies [10,11]. Before reaching the silicon substrate, incident light must propagate through a multilayer dielectric stack deposited during the CMOS fabrication process [1,11,12], consisting primarily of passivation layers, which protect the overall device from environmental contamination and degradation [6], and inter-metal dielectric (IMD) layers that provide electrical insulation between successive metal interconnections, preventing short-circuiting.

**Figure 1:**
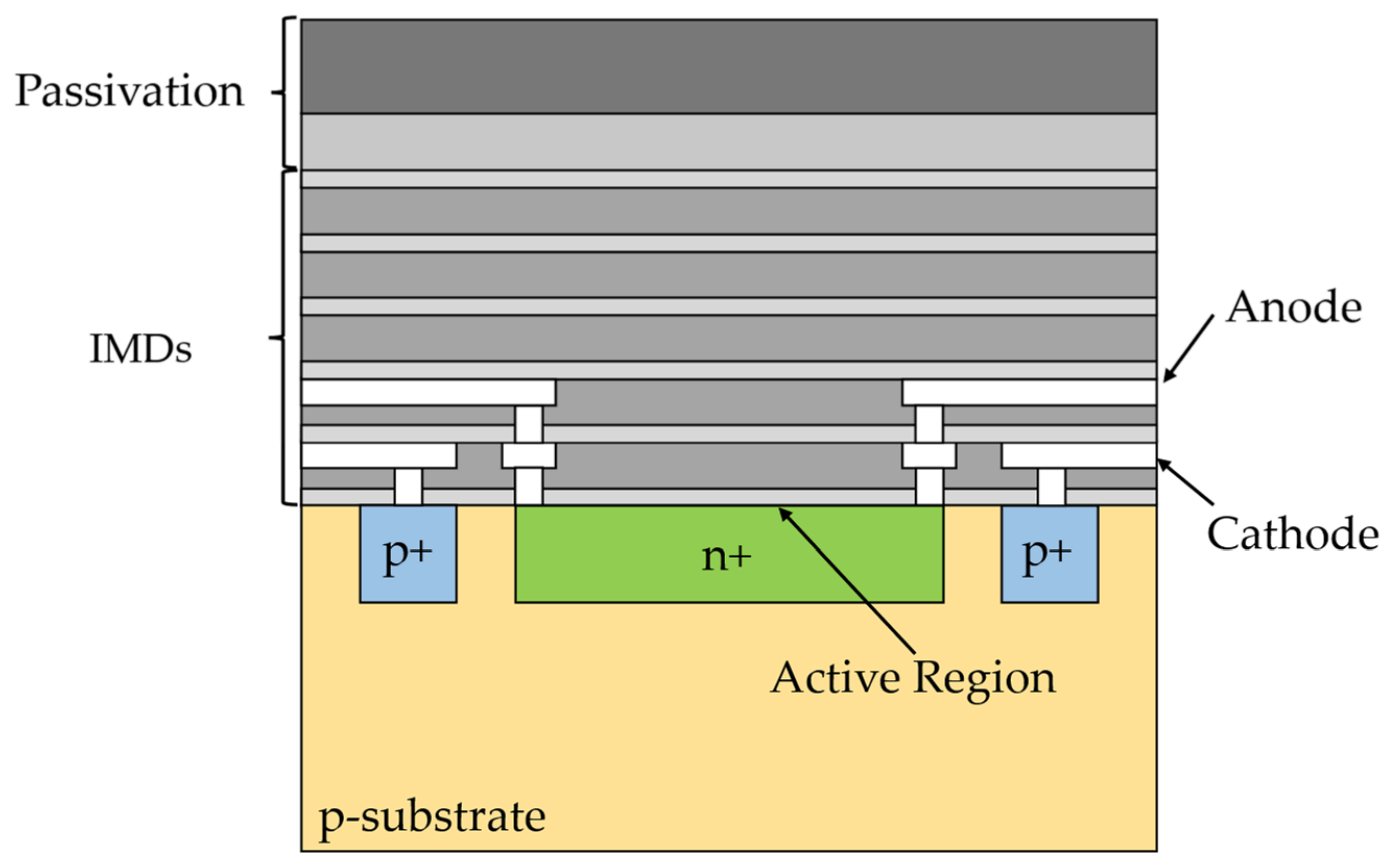
Representative cross-sectional schematic of a CMOS multilayer dielectric stack deposited above a n+/p-substrate vertical silicon photodiode.

Figure 1 illustrates a representative CMOS photodiode with the multilayer dielectric stack on top. As the optical wave propagates through the different dielectric layers, each interface partially reflects and transmits the incident light according to the refractive index mismatch between adjacent materials [13]. The optical waves reflected at successive interfaces accumulate different phase shifts while propagating through the dielectric layers. Their recombination produces constructive and destructive interference depending on the wavelength, refractive indices, layer thicknesses, and angle of incidence [9,11,13]. As a result, the optical power reaching the silicon active region may differ significantly from the incident optical power [9], thus directly influencing the responsivity and quantum efficiency of the photodiode [1].

Since these dielectric stacks are intrinsic to the CMOS fabrication process, they cannot be modified during the photodiode’s design. Therefore, accurately modelling their optical behavior becomes essential for predicting photodiode performance before fabrication [1,9]. This challenge becomes increasingly significant as CMOS technologies continue to scale down. For example, while the dielectric stack of the TSMC (Taiwan Semiconductor Manufacturing Company) 180 nm process consists of 14 dielectric layers distributed among four different materials with different refractive indexes (1.92 ≤ *n* ≤ 2.81) [14], the corresponding TSMC 65 nm technology contains 44 dielectric layers composed of five different materials (1.70 ≤ n ≤ 2.85) [15], primarily due to the increased number of metal interconnect layers. This larger number of dielectric interfaces increases the number of reflections experienced by the incident optical wave, producing considerably more complex interference patterns and making accurate numerical modelling increasingly challenging.

Several numerical approaches have been employed to model light propagation through multilayer optical structures, including the transfer-matrix method (TMM) [16], finite element full-wave electromagnetic simulations (FEM) [17], finite-difference time-domain (FDTD) methods [18], rigorous coupled-waves analysis (RCWA) [19], and ray-tracing techniques [20]. The selection and application of these methods depend on the physical assumption adopted for the thin-film structure, including the material properties, layer geometry, incidence conditions, and polarization of the incident light [21]. Each approach presents distinct advantages regarding physical accuracy, computational cost, and versatility. Consequently, selecting an appropriate numerical approach requires balancing physical accuracy with computational efficiency, since unnecessarily complex models may substantially increase simulations time without improving predictive accuracy, whereas overly simplified models may fail to capture relevant optical phenomena. While full-wave electromagnetic methods provide the most rigorous solution to Maxwell’s equations, simplified approaches such as TMM and thin-film ray optics can considerably reduce computational requirements for planar multilayer structures [22,23,24].

Although these numerical approaches have been extensively employed for the analysis of multilayer optical structures, most studies focus on a single modelling technique or a specific optical device [22,25]. Direct comparisons between different numerical approaches applied to realistic CMOS multilayer dielectric stacks remain limited, particularly regarding their relative accuracy, computational efficiency, and sensitivity to fabrication tolerances. Moreover, to the best of the authors’ knowledge, comparative studies investigating the optical transmission into the silicon substrate for both TSMC 65 nm and TSMC 180 nm CMOS dielectric stacks have not yet been reported. Such analyses are essential for the design and optimization of CMOS photodiodes and other integrated optical sensing devices fabricated using these technologies.

Accordingly, this work presents a comparative analysis of three numerical approaches for modelling the optical transmission through planar multilayer dielectric stacks representative of the TSMC 65 nm and TSMC 180 nm CMOS technologies. Wave Optics and Ray Optics simulations implemented in COMSOL Multiphysics, version 5.3, are compared with the TMM implemented in TFCalc. The comparison is performed through the spectral transmittance analysis over the 400 nm to 1000 nm wavelength range, sensitivity to process-induced thickness variations, incident light angular response, computational performance and model agreement. To ensure a fair comparison, all numerical approaches are applied to identical dielectric stack configurations and evaluated using the same material properties and illumination conditions. Thus, the aim of this work is not to introduce a new numerical formulation, but rather to assess whether computationally simpler approaches can reproduce the optical transmission of realistic CMOS dielectric stacks with sufficient accuracy for photodiode design, and to identify the conditions and geometrical assumptions under which each modeling approach is appropriate. The presented comparison therefore provides practical guidelines for selecting the most appropriate modelling strategy according to the required balance between physical accuracy, computational efficiency and geometrical complexity, which has not been previously reported in the literature.

## 2. Numerical Methods

To comprehensively evaluate and compare the optical transmission through the CMOS dielectric stacks, three distinct numerical approaches were employed: Wave Optics (FEM) [26] and Ray Optics (ray-tracing) [27], both implemented in the COMSOL Multiphysics software, and the TMM implemented with the TFCalc software. Because these modelling strategies rely on fundamentally different physical principles, ranging from rigorous electromagnetic field solving to geometrical ray tracing and matrix-based thin-film calculations, establishing a strictly standardized testing environment was crucial. This uniform baseline ensures that any observed variations in the simulated transmittance are directly attributed to the numerical and mathematical formulations of the models, rather than discrepancies in the input parameters.

For the standard configuration, the optical propagation was simulated over the 400 nm to 1000 nm wavelength range, covering the visible and near-infrared (NIR) spectrum relevant to standard silicon photodiode operation. The incident illumination was defined as unpolarized light to closely emulate practical light sources. Furthermore, these baseline evaluations assumed a perfectly normal angle of incidence across the average layer thickness. This specific setup served as the reference state for all three models before introducing further complexities, such as incident angle variations and process-induced thickness tolerances.

It is important to note that due to proprietary foundry restrictions of the two standard CMOS technologies (TSMC 65 nm and TSMC 180 nm), the exact materials of their dielectric layers are not disclosed. Consequently, the materials within both stacks are modelled based exclusively on their provided dielectric constants and corresponding refractive indices (*n*), which provide the necessary parameters for accurate optical simulation. Additionally, optical absorption of light within these layers was neglected, as the information on the extinction coefficient (*κ*) values of the individual materials was not provided by the foundry, specifying only that they exhibit low *κ* [14,15].

**Figure 2:**
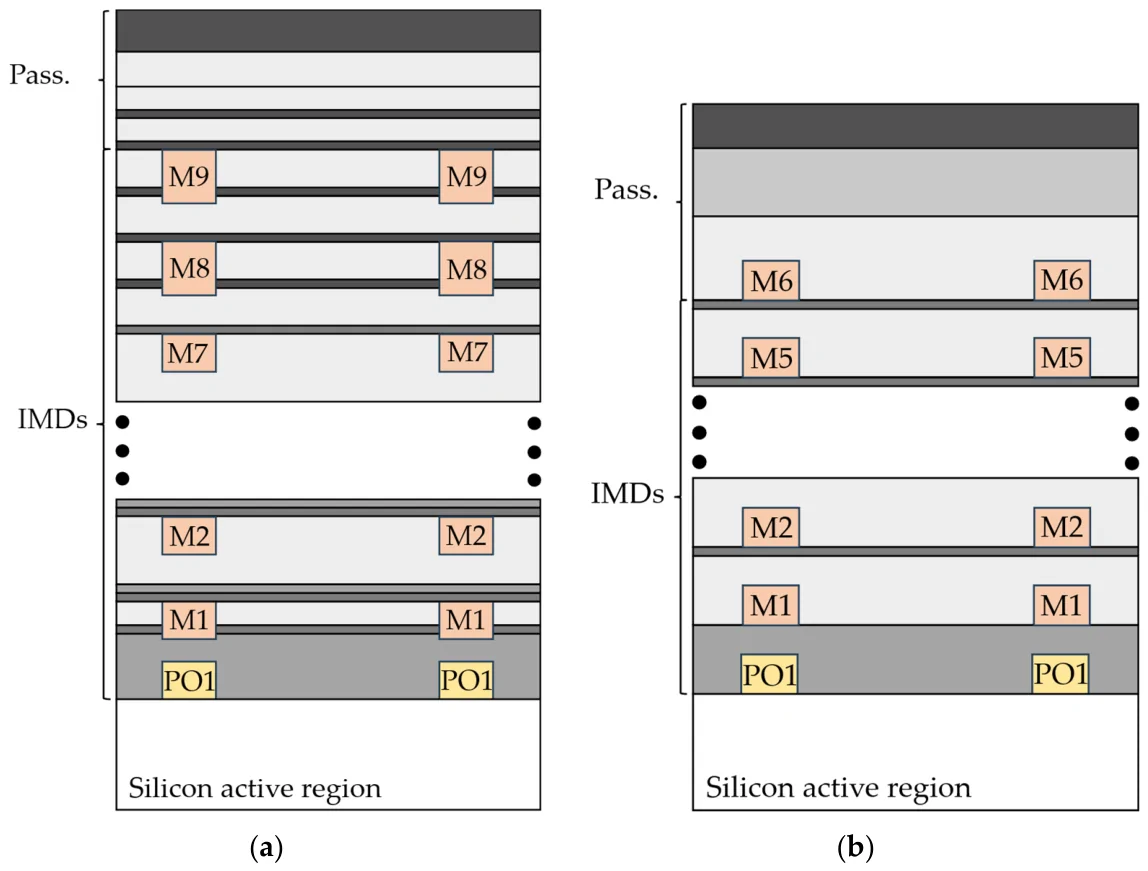
Simplified cross-sectional representations of the CMOS stack for the (**a**) TSMC 65 nm and (**b**) TSMC 180 nm technology, showing the passivation (Pass.) and IMD layers in the different gray colors, the metal layers (Mx) in orange and polysilicon (PO1) in yellow. The exact material and thickness of each layer are omitted due to confidentiality restrictions [14,15].

The TSMC 65 nm technology features a highly complex stack of 44 dielectric layers composed of five different materials, with refractive indices ranging from 1.70 to 2.85. The specific optical parameters used for this node are detailed in Table 1, while a simplified cross-sectional schematic is presented in Figure 2a [15].

Conversely, the TSMC 180 nm technology comprises a simpler stack of 14 dielectric layers distributed among four distinct materials, with refractive indices between 1.92 and 2.81. The material properties for this technology are summarized in Table 2, with a simplified cross-sectional representation in Figure 2b [14].

Finally, to complete the standard configuration, the underlying silicon substrate was modelled consistently across all three approaches. The wavelength-dependent complex refractive index of intrinsic silicon was defined using the well-established empirical data provided by Green and Keevers [28].

### 2.1. Wave Optics

**Figure 3:**
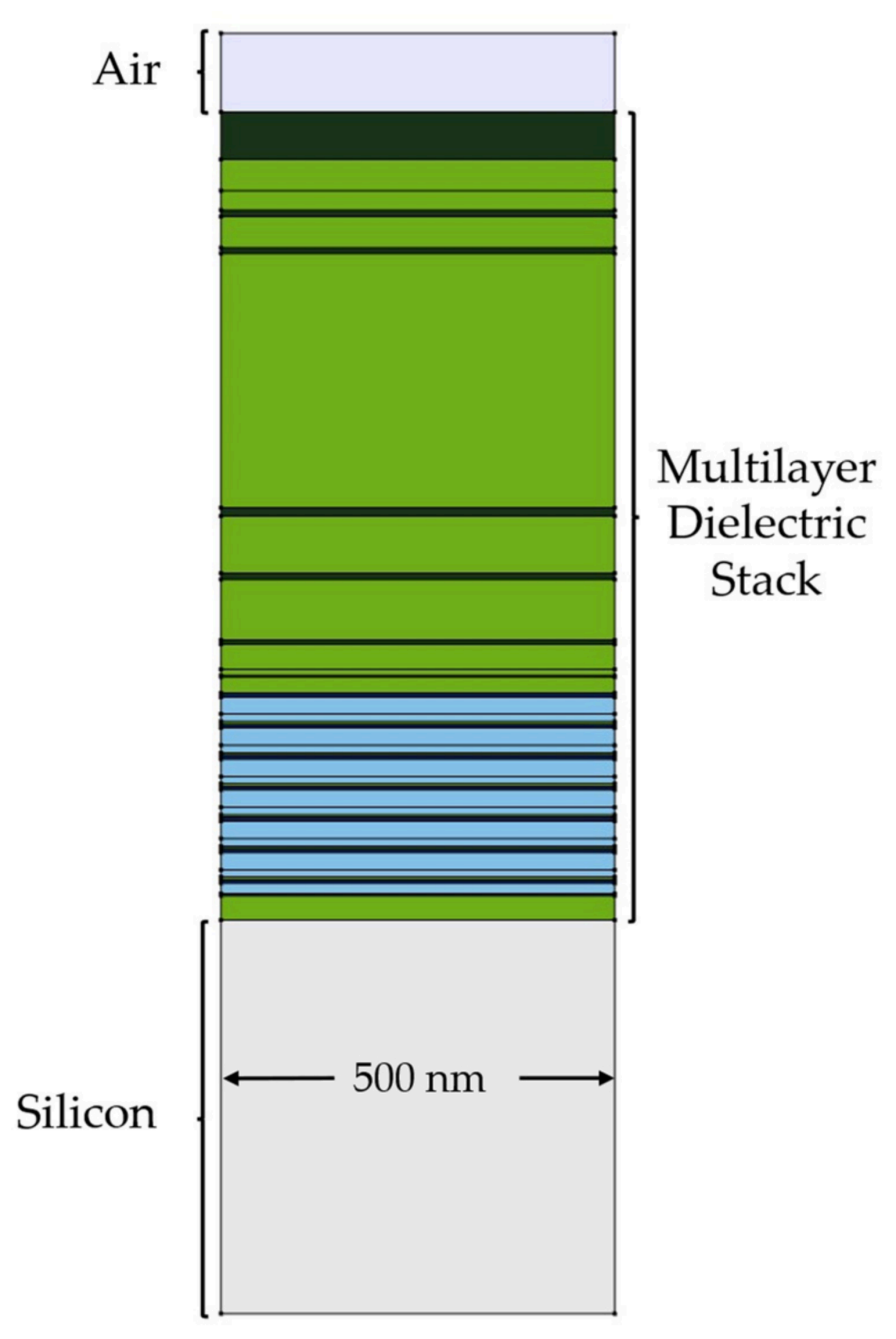
Two-dimensional geometry for the Wave Optics model, illustrating the air domain on top, the intermediate multilayer dielectric stack, and the silicon substrate, using the TSMC 65 nm technology stack.

COMSOL Multiphysics software, version 5.3, was used to simulate the optical transmission through the multilayer dielectric stack. Since the stack is laterally uniform and extends in the transverse directions, optical propagation can be accurately represented using a simpler 2D geometric domain. This assumption significantly reduces the computational cost while preserving the relevant physical phenomena governing the transmission through the stack. The model geometry is shown in Figure 3, with a width of 500 nm and divided into three main regions: an air domain at the top, the intermediate multilayer dielectric stack in the middle, and the silicon substrate at the bottom. Notice that each color represents a different material from the stack for easier distinction and identification.

The numerical model evaluates the propagation of electromagnetic waves using the Electromagnetic Waves, Frequency Domain interface. The full-field propagation through the different media, derived from Maxwell’s equations, is governed by the frequency-domain wave equation [29]:

(1) $$\nabla \times \mu_{r}^{-1}\left(\nabla \times E\right)-k_{0}^{2}\left(\epsilon_{r}-\frac{\mathrm{j\sigma}}{\omega \epsilon_{0}}\right)E=0$$

(2) $$k_{0}=\frac{2\pi }{\lambda_{0}}$$

where ***E*** is the electric field vector, *μ_r_* is the relative permeability of the medium, *k*_0_ is the free-space wavenumber, *ϵ_r_* is the relative permittivity, *σ* is the electrical conductivity, *ω* is the angular frequency, *ϵ*_0_ is the vacuum permittivity, and *λ_0_* is the vacuum wavelength.

To describe the optical behavior of the layers, the materials were defined using the complex refractive index formulation. The relative permittivity is given by [30],

*ϵ_r_* = (*n* − *iκ*)^2^ (3)

with the electrical conductivity set to 0 and the relative permeability to 1 across all domains. The model incorporates the following boundary conditions to emulate an infinitely periodic planar structure:

1. Floquet periodic boundary conditions were applied to the lateral boundaries of the 2D domain. This periodicity enforces a phase shift between the source (*src*) and destination (*dst*) boundaries, governed by [26]:
  (4) $$E_{\mathrm{dst}}=E_{\mathrm{src}}e^{-ik_{F}\cdot \left(r_{\mathrm{dst}}-r_{\mathrm{src}}\right)}$$
  (5) $$H_{\mathrm{dst}}=H_{\mathrm{src}}e^{-ik_{F}\cdot \left(r_{\mathrm{dst}}-r_{\mathrm{src}}\right)};$$
2. A periodic port (Port 1) was assigned to the top air boundary to inject the incident wave into the system;
3. A passive periodic port (Port 2) was placed at the bottom silicon boundary to absorb the outgoing wave, preventing back-reflections into the domain.

Assuming negligible optical absorption within the dielectric thin films, the overall spectral transmittance reaching the silicon substrate (*T_Si_*) was determined indirectly via the conservation of energy. As the total optical reflectance (*R_tot_*) and transmittance (*T_tot_*) are given at the specific input and output ports (respectively, Port 1 and Port 2), and Port 2 prevents reflection at the bottom boundary, it is valid to assume that the optical power reflected back into Port 1 is the power that does not enter the silicon region. Consequently, the transmittance into the silicon substrate can be calculated as,

*T_Si_* = 1 − *R_tot_* (6)

As most laboratory light sources are essentially unpolarized, the model was solved twice for each wavelength, once for Transverse Magnetic (TM) polarization and once for Transverse Electric (TE) polarization. For TM polarization, the incident magnetic field was defined strictly out-of-plane, while for TE polarization, the same was done for the incident electric field. The final unpolarized spectral transmittance was then obtained by averaging the transmittances from both polarizations. Although the TE and TM responses are identical under normal incidence, both polarization states were considered to ensure consistency with the angular response analysis, where polarization-dependent effects become significant.

The 2D domain was discretized using a physics-controlled mesh set to a normal element size, which adapts the mesh resolution based on the material’s refractive index and the operating wavelength to ensure numerical accuracy. Table 3 presents the number of elements and average quality of the mesh for the two CMOS technologies.

Two distinct studies were performed to evaluate the stacks, both utilizing the MUMPS (MUltifrontal Massively Parallel Sparse) direct solver to compute the electromagnetic fields. The first study focused on spectral transmittance, employing a parametric study to vary the incident free-space wavelength from 400 nm to 1000 nm (in steps of 0.5 nm) at normal incidence. The second study evaluated the incident light angular response at a fixed reference wavelength of 450 nm (selected as an illustrative case within the visible spectrum that aligns with target wavelengths of interest in integrated optical sensing applications) by performing an auxiliary sweep of the incident angle from 0° to 80° (in steps of 0.5°).

### 2.2. Ray Optics

**Figure 4:**
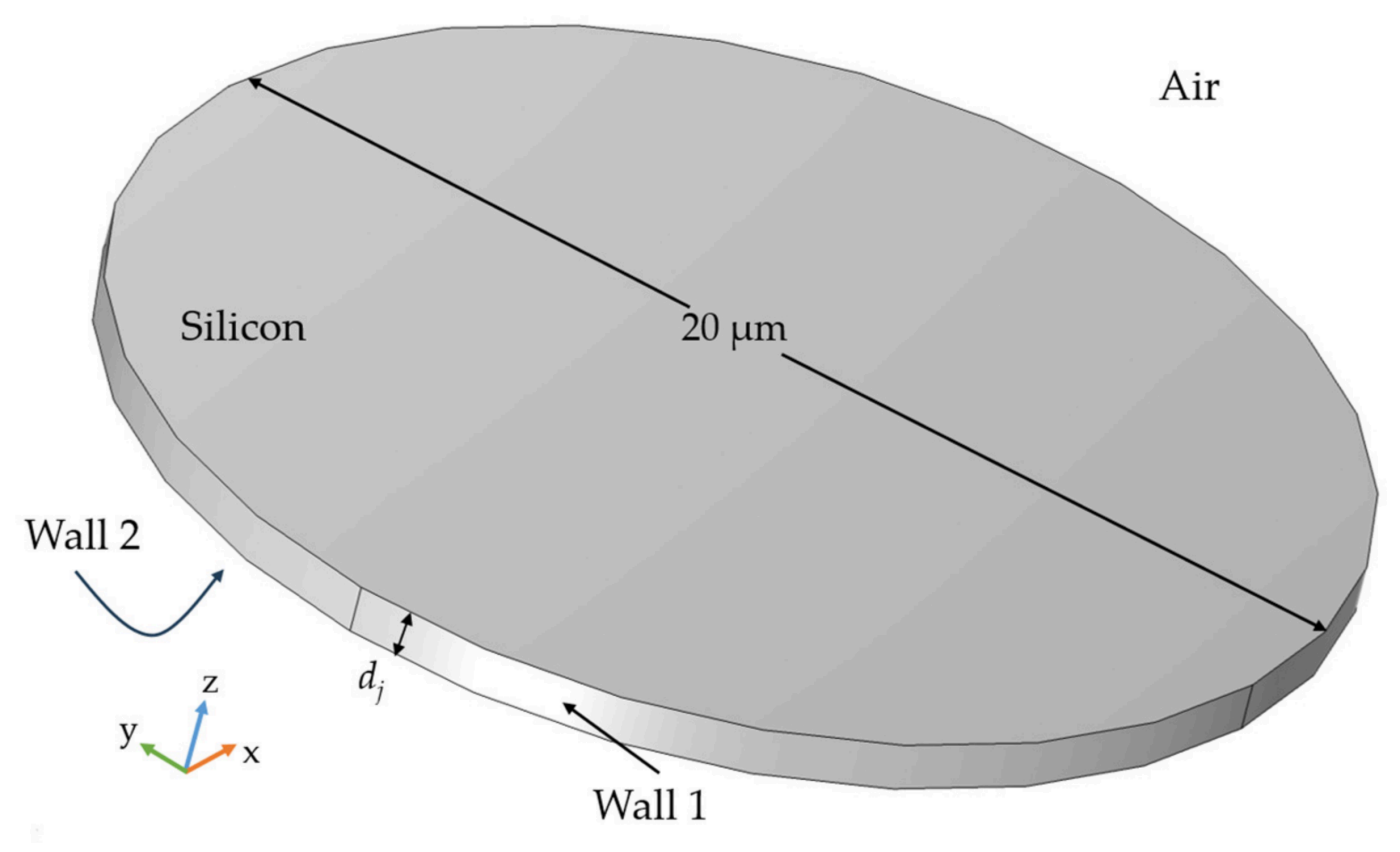
Three-dimensional cylindrical geometry for the Ray Optics model, consisting of the exterior air domain and the silicon substrate.

COMSOL Multiphysics software, version 5.3, was also used to implement the Ray Optics approach, evaluating optical propagation through the Geometrical Optics interface. Unlike the Wave Optics model, this approach utilized a 3D cylindrical geometric domain, with a diameter of 20 μm and a height of 1.6 μm, as presented in Figure 4. However, explicitly drawing the complete multilayer stack within this 3D environment is neither practical nor consistent with the assumptions of geometrical optics. A fundamental limitation of classical geometrical optics is the assumption that physical structures must be significantly larger than the wavelength of the incident light. In particular, conventional geometrical ray tracing does not intrinsically reproduce coherent thin-film interference in sub-wavelength layers. To overcome this limitation and accurately model the layers of the CMOS stack, the thin dielectric films were not drawn in the model geometry. Instead, the geometric domain consisted solely of air, as exterior domain, and the silicon substrate, with the films represented analytically at their interface through equivalent reflection and transmission coefficients.

The trajectory of the optical rays through the domain is determined by solving the Hamiltonian ray equations [31],

(7) $$\frac{dq}{\mathrm{dt}}=\frac{\partial \omega }{\partial k}$$

(8) $$\frac{dk}{\mathrm{dt}}=-\frac{\partial \omega }{\partial q}$$

where ***q*** is the position vector and ***k*** the wave vector. While the ray propagation itself is treated using conventional geometrical optics, the thin-film interference effects of the omitted multilayer CMOS stack were recovered analytically at the air-silicon boundary using a Material Discontinuity node. This feature computes the equivalent reflection (*r_eq_*) and transmission (*t_eq_*) coefficients for the entire multilayer stack using analytical thin-film interference equations [32,33]:

(9) $$r_{\mathrm{eq}}=\frac{r_{1}+r_{2}e^{2\mathrm{i\beta}}}{1+r_{1}r_{2}e^{2\mathrm{i\beta}}}$$

(10) $$t_{\mathrm{eq}}=\frac{t_{1}t_{2}e^{\mathrm{i\beta}}}{1+r_{1}r_{2}e^{2\mathrm{i\beta}}}$$

(11) $$\beta =\frac{2\pi }{\lambda_{0}}\mathrm{nt}\cos\theta$$

where *r_1_* and *t_1_* represent the Fresnel reflection and transmission coefficients, respectively, at the first interface, *r_2_* and *t_2_* are the corresponding coefficients at the second interface, *β* is the phase accumulated during propagation through the dielectric film, *t* is the physical thickness of the film, and *θ* is the propagation angle inside the film. Therefore, although the thin dielectric layers are not explicitly resolved in the ray-tracing geometry, their optical interference is incorporated through the analytical Material Discontinuity node.

The model incorporates specific boundary conditions and release features to simulate the optical source and the domain limits:

1. The incident light was generated using a Release from Grid node. The initial polarization was explicitly set to unpolarized, and rays were released with a polychromatic wavelength distribution ranging from 400 nm to 1000 nm.
2. The initial intensity of the released rays was normalized to 1 W/m^2^. The initial propagation direction was defined by the incident angle *α*, mapped to the direction vector ***L***_0_ = (0, sin(*α*), −cos(*α*)).
3. For the domain boundaries, the outer cylindrical wall (Wall 1) was configured with a Freeze condition to stop rays from propagating outside the region of interest.
4. The bottom of the silicon substrate (Wall 2) was set to Pass through, allowing rays to exit the simulation domain freely without reflecting.

To determine the optical transmittance into the silicon substrate, the intensity and phase of the rays were explicitly computed during propagation. For simplicity, with the initial optical power of the released rays set to 1 W/m^2^, the final intensity of the rays immediately after entering the silicon directly corresponds to the fraction of optical power successfully transmitted into the substrate.

The 3D domain was discretized using a physics-controlled mesh set to a normal element size. Given that the dielectric layers were replaced by the Material Discontinuity boundary condition, the defined mesh size was sufficient to accurately resolve the macroscopic air and silicon domains without unnecessary computational overhead. For the two CMOS technologies, as the model geometry does not change, they both present a mesh with 1120 elements.

To determine the optical transmittance, two studies were performed, using the MUMPS solver again. The first utilized a Ray Tracing study step, configured with intervals of 10^−17^ s to observe the ray travel around 1 nm per iteration. Since the primary objective was to extract the transmitted intensity at the exact moment the rays entered the silicon substrate, the maximum tracing time was kept just long enough for the rays to successfully cross the dielectric interface and enter the silicon. The second study evaluated the angular response by performing a Parametric Sweep of the incident angle from 0° to 80° (0.5° steps).

### 2.3. TFCalc

The third approach used to evaluate the optical transmission was the TFCalc thin-film optics software, version 3.5, which implements the TMM. Unlike the previous COMSOL-based approaches, TFCalc does not rely on a drawn spatial domain. Instead, the multilayer structure is defined mathematically as a 1D array of physical thicknesses and refractive indices.

The software computes the optical response using the characteristic matrix formulation, where each layer *j* is represented by a 2 × 2 matrix *M_J_* [34],

(12) $$M_{j}=\left[\begin{array}{cc} \cos\left(\beta_{j}\right) & \frac{i}{\eta_{j}}\sin\left(\beta_{j}\right)\\ i\eta_{j}\sin\left(\beta_{j}\right) & \cos\left(\beta_{j}\right) \end{array}\right]$$

where *β_j_* represents the phase accumulated during propagation through the dielectric film, analogous to the formulation defined in Equation (11), and *η_j_* is the optical admittance. The total system matrix *M*, for *m* amount of layers, is obtained by multiplying the individual layer matrices [34]:

(13) $$M=\prod_{j=1}^{m}M_{j}$$

**Figure 5:**
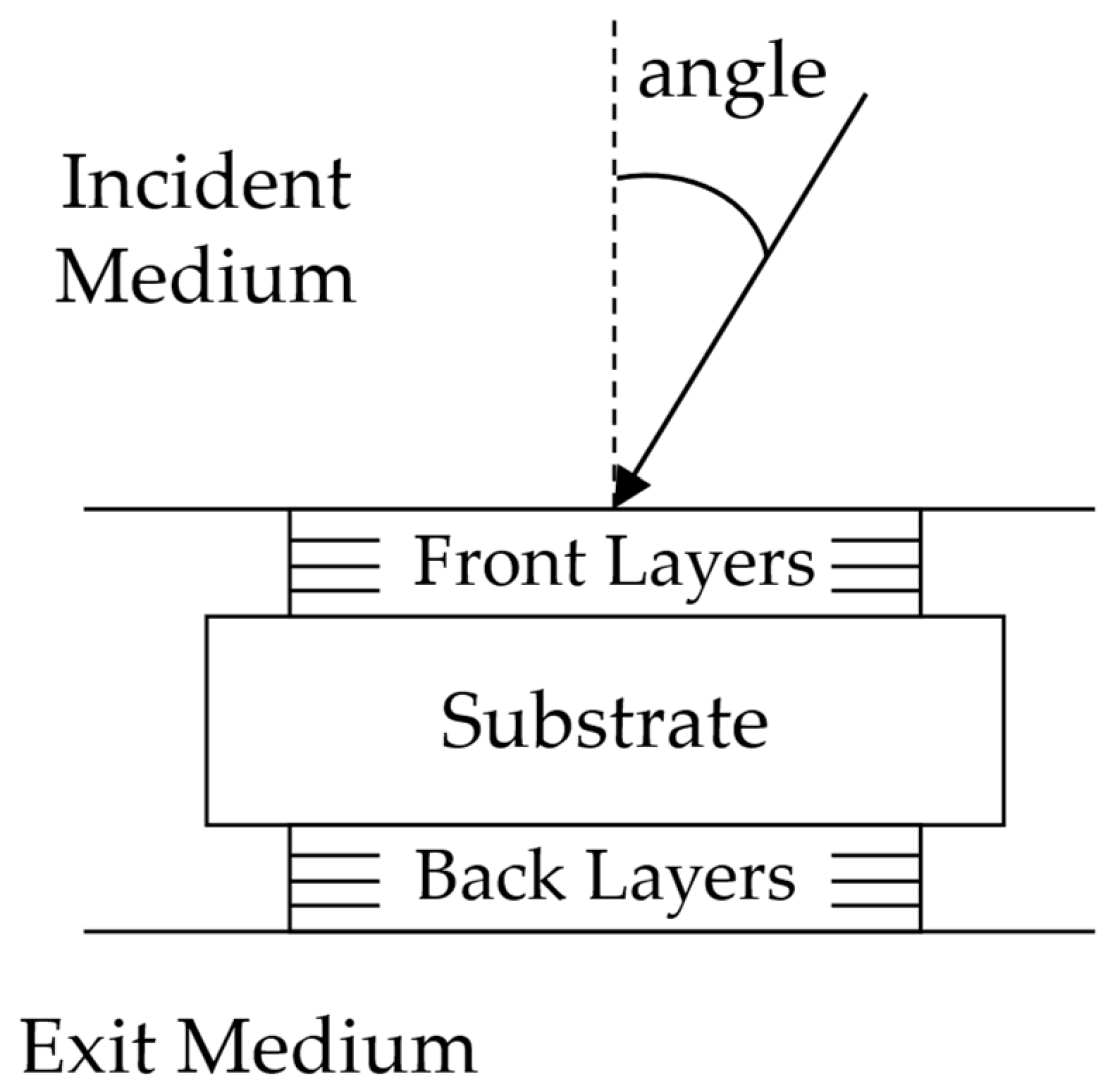
Schematic representation of the TFCalc physical environment model.

The physical configuration conceptually divides the model into distinct regions, as shown in Figure 5. The incident and exit media were defined as air, while the intermediate structure was set as a silicon substrate with a bulk substrate thickness of 0.5 mm. In TFCalc, the substrate is treated inherently as a massive medium that supports optical attenuation but does not generate thin-film interference within its volume. The exact sequences for the TSMC 65 nm and TSMC 180 nm technologies were entered into the Front Layers region, while no Back Layers were utilized.

To match the previous numerical models, the analysis utilized a WHITE illuminant, an IDEAL detector (100% efficiency), and calculated the average of the P-polarization (TM) and S-polarization (TE) states to simulate unpolarized light. Two primary sweeps were executed within the Set Analysis Parameters Interface, a wavelength sweep from 400 nm to 1000 nm (in steps of 0.5 nm) at normal incidence, and an incident angle sweep from 0° to 80° (in increments of 0.5°) at the fixed reference wavelength of 450 nm.

## 3. Results and Discussion

This section presents a comprehensive evaluation of the optical transmission through the TSMC 65 nm and TSMC 180 nm dielectric stacks. The analysis begins with a baseline comparison of the spectral transmittance derived from the three numerical approaches. The following studies investigate the impact of process-induced thickness tolerances, evaluate the incident light angular response to emulate practical illumination conditions, and finally, assess the computational efficiency and agreement of each modelling technique.

### 3.1. Baseline Spectral Transmittance

**Figure 6:**
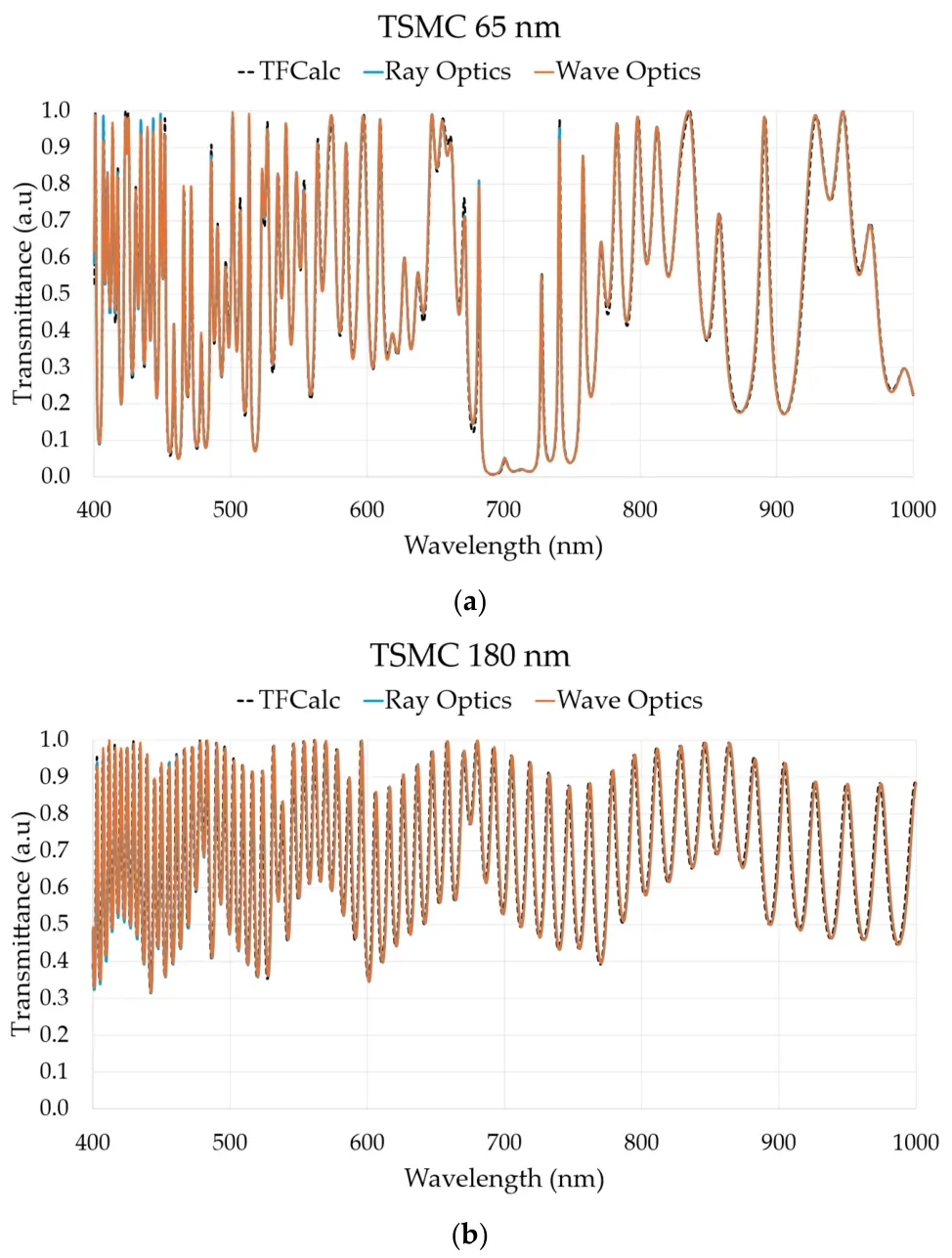
Spectral transmittance at normal incidence for the (**a**) TSMC 65 nm and (**b**) TSMC 180 nm dielectric stacks, comparing the Wave Optics (solid orange line), Ray Optics (solid blue line) and TFCalc (dashed black line).

The baseline optical transmittance of the TSMC 65 nm and TSMC 180 nm CMOS technologies was evaluated at normal incidence from 400 nm to 1000 nm. The three numerical approaches exhibit excellent overall agreement across the evaluated spectrum, with no major discrepancies between their predictions, as presented in Figure 6. A direct comparison between the two CMOS nodes reveals distinct optical behavior dictated by their physical structures. The TSMC 65 nm technology produces a highly complex optical transmittance profile characterized by rapid oscillations and large fluctuations across the entire transmittance range, as shown in Figure 6a. These high-amplitude variations directly correspond to its more complex dielectric stack, which features a higher layer count and numerous different refractive index interfaces. The larger number of dielectric layers increases the number of partial reflections and accumulated path differences, resulting in a denser interference pattern and consequently more rapid transmittance oscillations. The TSMC 180 nm technology presents a significantly more uniform curve and maintains a generally higher transmittance, never going below 31% across the entire wavelength range.

**Figure 7:**
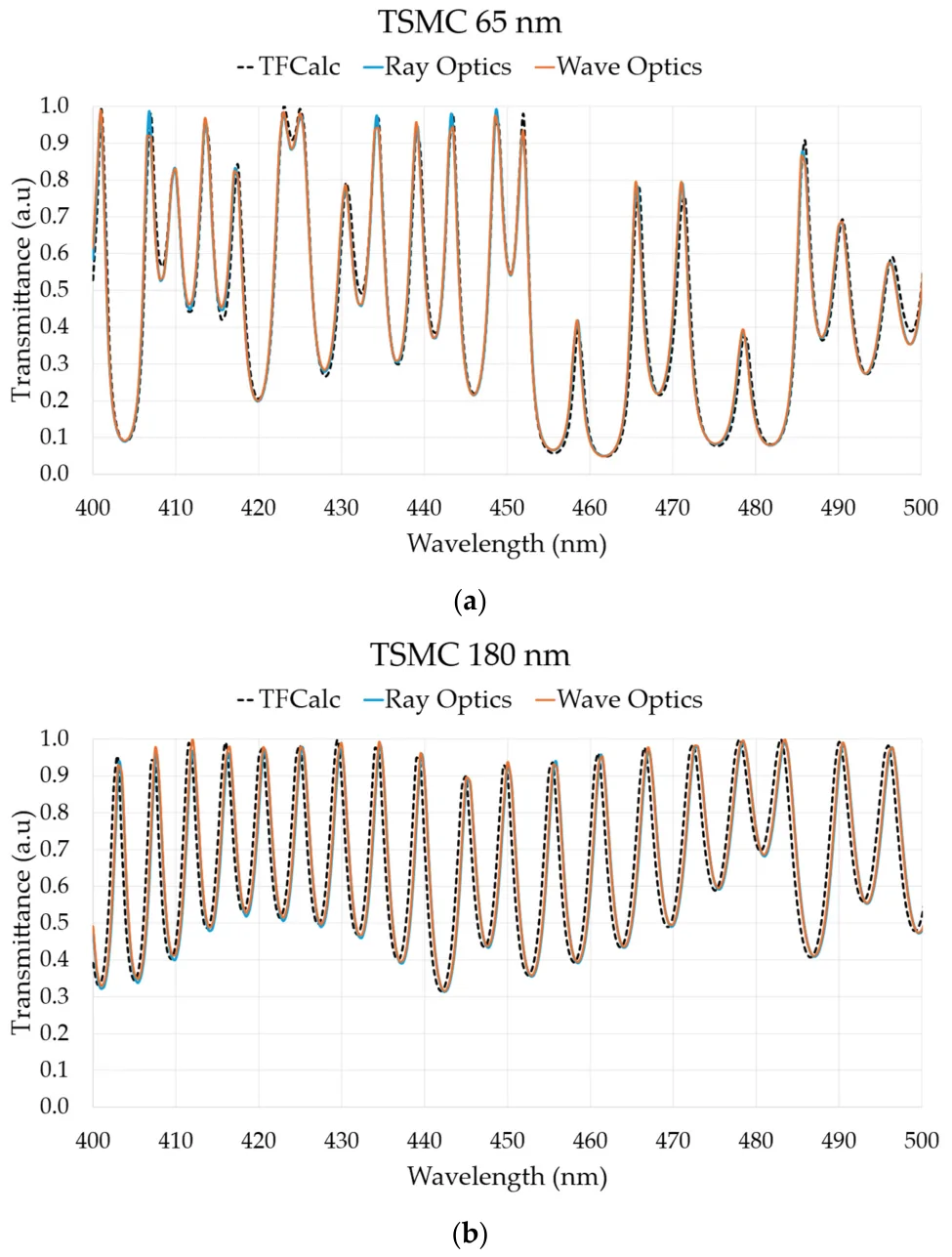
Detailed view of the spectral transmittance in the 400 nm to 500 nm range for the (**a**) TSMC 65 nm and (**b**) TSMC 180 nm technologies.

A closer examination of the curve, for example, looking at the 400 nm to 500 nm spectral region reveals minor quantitative variations among the models. As represented by the dashed black lines in Figure 7, for both CMOS technologies, the TFCalc model results exhibit a slight wavelength shift relative to the two COMSOL models. This small spectral offset represents the primary deviation observed among the three computational approaches under strictly normal incidence conditions, together with small, localized deviations, specifically at the smaller wavelengths. Notably, this discrepancy is slightly more pronounced for the TSMC 180 nm technology. Since the Ray Optics results show very close agreement with the full-wave FEM model, this observed offset in TFCalc is unlikely to originate from the simplified description itself. Instead, it is more likely related to differences in the numerical implementation of the transfer-matrix calculation between TFCalc and COMSOL. Although the TSMC 180 nm stack features fewer dielectric layers compared to the TSMC 65 nm stack, its individual layers are generally thicker, resulting in larger optical path lengths. Consequently, small differences in the calculated phase accumulation or refractive index representation may produce a more noticeable shift in the resulting interference pattern for the 180 nm stack. Thus, the lower number of layers does not necessarily imply a smaller discrepancy between the numerical models.

### 3.2. Process Robustness and Thickness Variation

Semiconductor foundries provide nominal layer thicknesses alongside statistical tolerance bounds of, in the case of TSMC, ±3% to ±20%, depending on the layer. To understand how these manufacturing variations affect the optical response, a process robustness study was conducted, where the thickness of each layer was changed to their minimum and maximum value according to the foundry. Since all three numerical approaches produced nearly identical transmittance profiles across these variations (see Supplementary Material, Figures S1 and S2), the Ray Optics results are utilized here to illustrate the fundamental spectral behavior.

**Figure 8:**
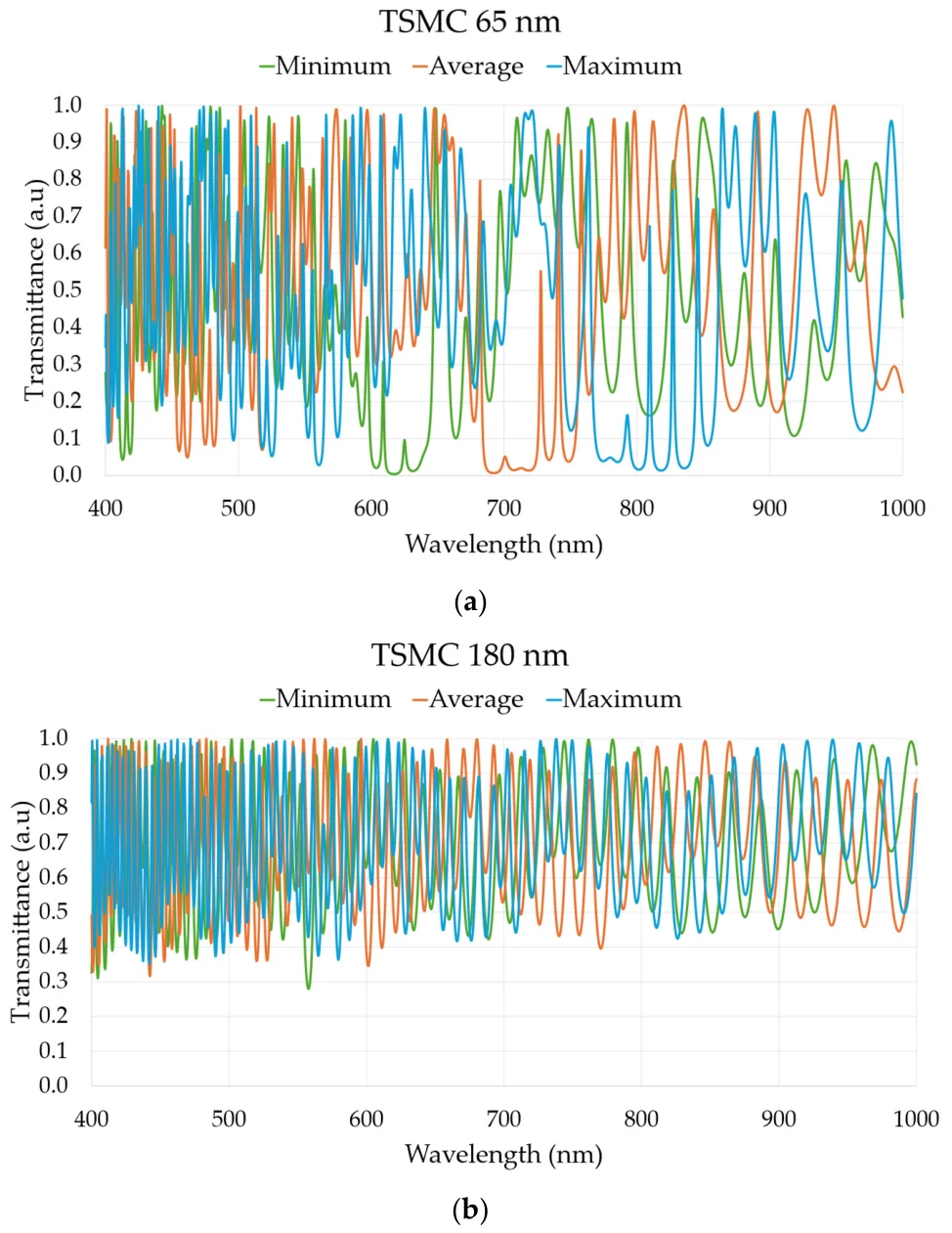
Impact of process induced thickness variations on the overall spectral transmittance for the (**a**) TSMC 65 nm and (**b**) TSMC 180 nm technologies, simulating the minimum (green line), average (orange line) and maximum (blue line) thicknesses.

**Figure 9:**
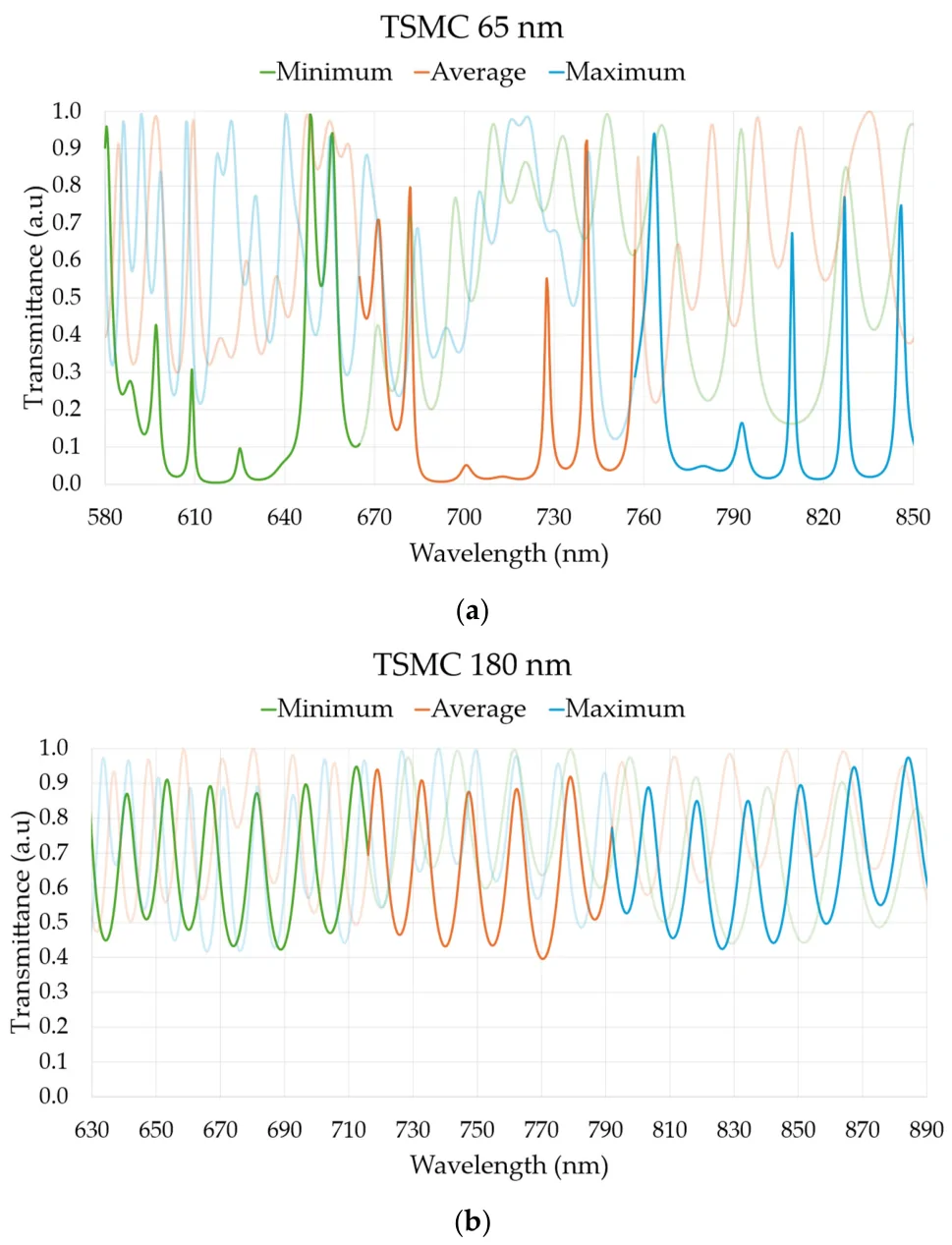
Zoomed in view of the spectral transmittance for the (**a**) TSMC 65 nm and (**b**) TSMC 180 nm technologies, detailing the horizontal wavelength shifts induced by the manufacturing thickness variations.

Varying the layer thicknesses to their manufacturing limits induces a distinct spectral shift in the overall transmittance curves, as shown in Figure 8. For both TSMC technologies, the minimum thickness results in a shift toward smaller wavelengths, whereas the maximum thickness results in a shift toward larger wavelengths. This displacement is particularly evident in the zoomed spectral regions presented in Figure 9, which specifically reveals a shift of approximately ±90 nm and ±80 nm, for TSMC 65 nm and TSMC 180 nm technologies, respectively. This behavior is expected because increasing the optical thickness of each layer increases the accumulated phase delay, shifting constructive and destructive interference towards longer wavelengths, whereas reducing the thickness produces the opposite effect.

These results indicate that while thickness variations do not significantly alter the interference patterns, they do shift the overall transmittance curve. Consequently, experimental characterization of fabricated photodiodes is expected to exhibit these potentially significant spectral shifts relative to nominal simulations, strictly reflecting the inherent CMOS process tolerances. For photodiodes designed to operate at a specific target wavelength, these shifts represent an important design risk, since a transmittance maximum or minimum predicted at the nominal layer thickness may be displaced away from the intended operating wavelength after fabrication. In the present case, shifts of approximately ±90 nm for TSMC 65 nm and ±80 nm for TSMC 180 nm demonstrate that this effect can be comparable to a substantial fraction of the visible spectrum.

Therefore, when optimizing a photodiode for a specific target wavelength, an appropriate wavelength margin should be considered during the design phase rather than relying exclusively on the nominal layer thickness. The target response should ideally remain within an acceptable transmittance range over the expected fabrication tolerance limits, reducing the sensitivity of the final device performance to process-induced thickness variations.

### 3.3. Incident Light Angular Response and Illumination Effects

The angular dependence of the multilayer dielectric stacks represents an important practical aspect of their optical performance. To assess this behavior, transmittance was simulated across an incident angle sweep from 0° to 80° at a fixed reference wavelength of 450 nm.

**Figure 10:**
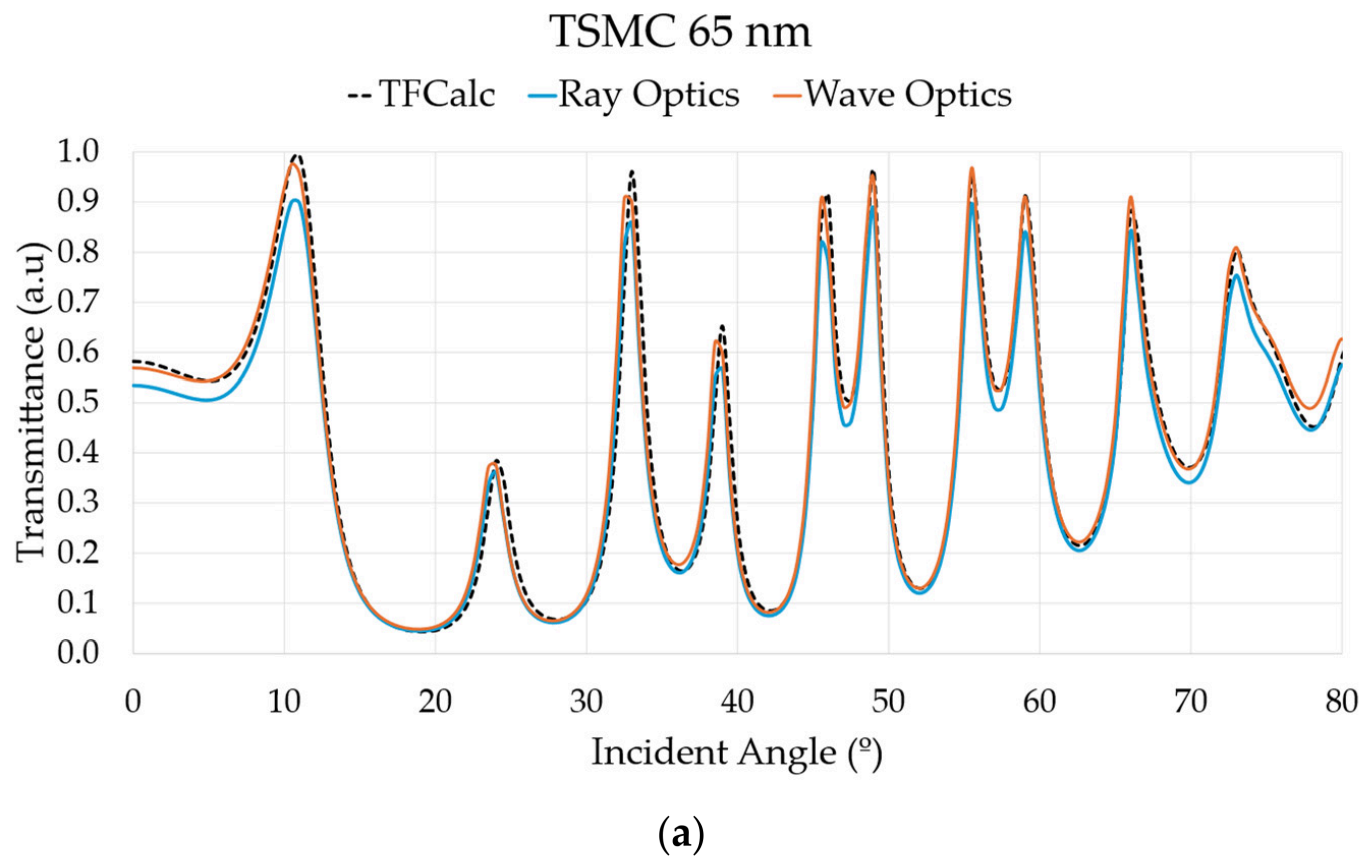

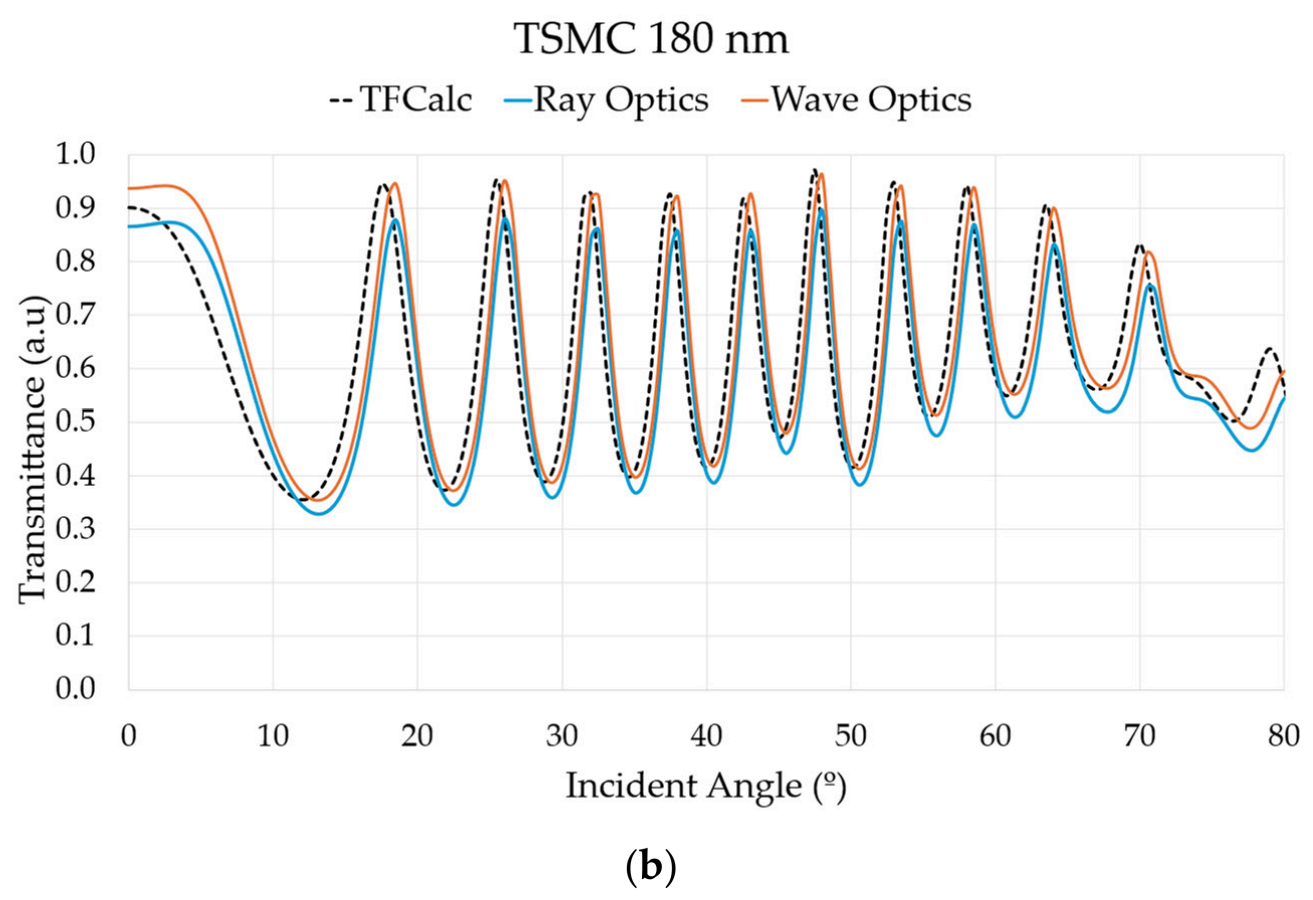
Angular transmittance response as a function of the incident angle from 0° to 80°, evaluated at a fixed wavelength of 450 nm, for the (**a**) TSMC 65 nm and (**b**) TSMC 180 nm technologies across the three numerical approaches.

As illustrated in Figure 10, all three models remain generally in agreement across the entire angular sweep. The results demonstrate strong, continuous transmittance oscillations as the angle of incidence increases. This high angular sensitivity has direct implications for practical laboratory testing. In experimental practice, photodiodes are frequently characterized using LEDs (Light-Emitting Diodes), which emit uncollimated light across a wide range of angles. Under these practical conditions, the photodiode receives light over a broad range of incident angles rather than receiving a single discrete normal incidence beam. Since the optical path length within each dielectric layer varies with the angle of propagation, the interference conditions continuously evolve as the incident angle increases [21].

**Figure 11:**
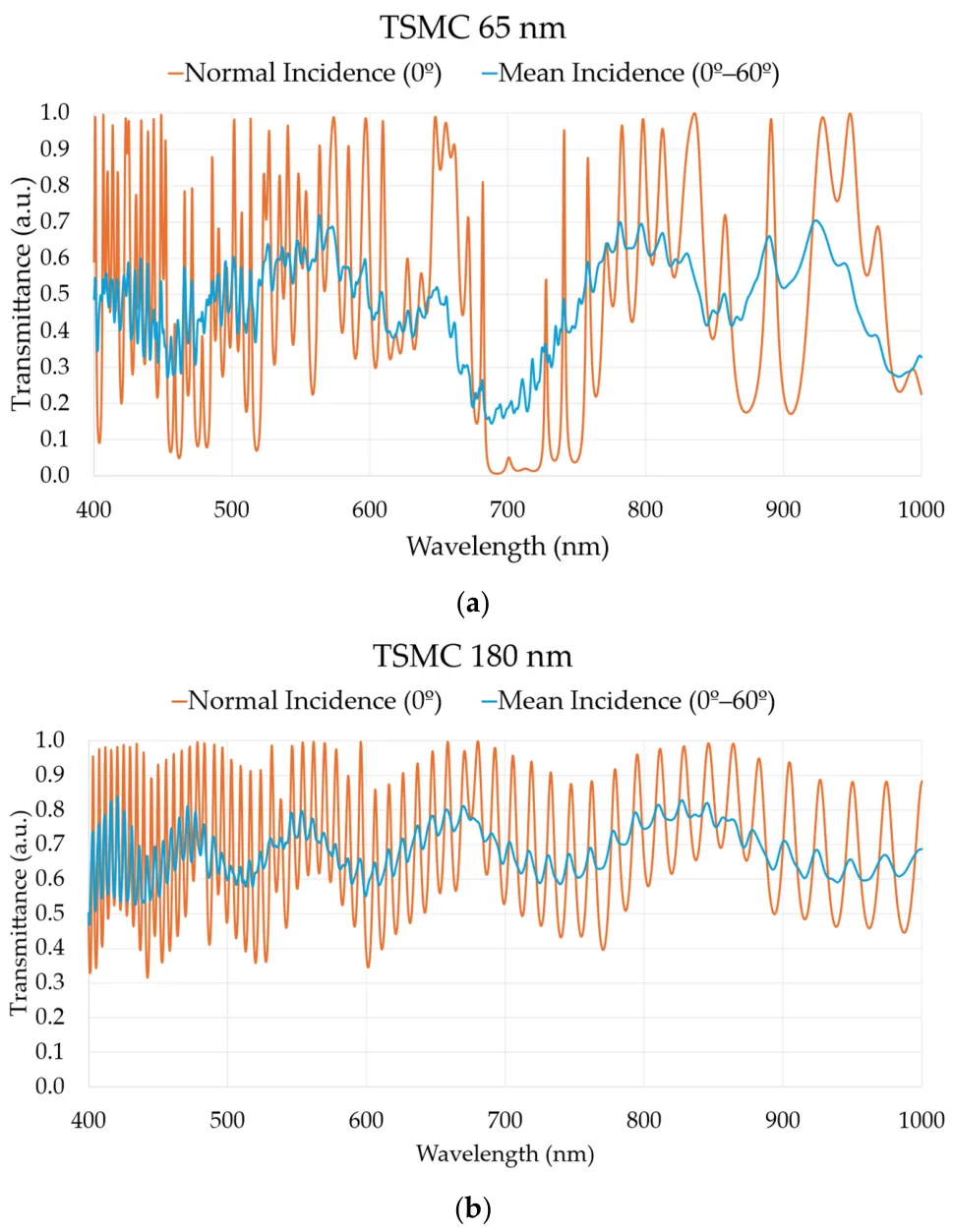
Comparison between the strictly normal incidence transmittance and the mean transmittance averaged over an incident angle cone of 0° to 60° for the (**a**) TSMC 65 nm and (**b**) TSMC 180 nm technologies, illustrating the smoothing effect of practical uncollimated illumination.

To quantify this illumination effect, an angular averaging study was performed to calculate the mean transmittance for incident angles from 0° to 60°. This averaging process dampens the interference effects, smoothing the overall spectral curve and significantly reducing the amplitude of the oscillations (Figure 11). Consequently, experimental characterizations utilizing standard uncollimated LED illumination are expected to produce a much smoother responsivity curve, effectively masking several of the sharp interference peaks predicted by ideal normal incidence simulations.

This smoothing effect is therefore dependent on the angular distribution of the illumination source and should not be considered a general characteristic of the photodiode response. In particular, for a collimated laser source, where the incident light is concentrated within a narrow angular range, the angular averaging effect is substantially reduced and the sharp interference features predicted under near normal incidence conditions are expected to remain observable. Consequently, the applicability of angular averaging as a representation of experimental illumination depends on the optical characteristics of the light source, with the present 0° to 60° averaging being most representative of uncollimated illumination such as that produced by the LED conditions considered here.

The angular averaging results also provide an important practical consideration for photodiode design. While process-induced thickness variations primarily produce a displacement of the interference features, angular averaging acts differently by reducing the amplitude variation in these features when the device is illuminated by a distribution of incident angles. Therefore, angular averaging can partially mitigate the sensitivity of the measured spectral response to narrow interference features under practical uncollimated illumination, but it does not compensate for the wavelength displacement caused by fabrication tolerances. The two effects should then be considered independently: process tolerance awareness is required to account for the uncertainty in the spectral position of the interference features, whereas angular averaging determines how strongly these features remain observable under realistic illumination conditions. For applications targeting a specific wavelength, this distinction is particularly relevant, since a design may be optimized to tolerate the expected fabrication-induced wavelength shift while the use of realistic illumination conditions can further reduce the sensitivity to sharp spectral variations.

### 3.4. Computational Efficiency and Model Agreement

A key objective of this comparative analysis was to evaluate both the computational efficiency and the agreement between the numerical approaches. Execution times were recorded for the baseline spectral transmittance study (wavelength sweep from 400 nm to 1000 nm, in 0.5 nm steps) of the TSMC 65 nm stack. Table 4 summarizes the computational times required to execute the full spectral analysis and the quantitative error metrics. The Wave Optics (FEM) model required nearly four hours to complete the computation. The Ray Optics model reduced this time drastically, completing the same study with very similar results in approximately forty seconds. The TFCalc model also provided very accurate results, when compared to the other models, while requiring approximately one second to completely compute.

While the Wave Optics model inherently provides the most comprehensive full-field electromagnetic information and greater geometric versatility, making it essential for complex, non-planar or 3D structures, it is highly computationally intensive. For the laterally uniform planar stacks considered in this work, the TFCalc and Ray Optics models deliver very similar transmittance results at a fraction of the computational cost. To quantitatively validate this agreement, the Mean Absolute Difference (MAD) and Mean Relative Difference (MRD) were calculated using the full-wave electromagnetic simulations as the reference baseline. The TFCalc model demonstrated a MAD of 2.25% and a MRD or 5.55%, while the Ray Optics model achieved an even closer alignment with a MAD of 0.49% and a MRD of 1.19%. These metrics confirm that these models successfully capture the complex thin-film interference behavior without significant accuracy degradation. Ultimately, the TMM approach implemented in TFCalc demonstrates the most favorable trade-off between accuracy and efficiency for analyzing planar dielectric stacks in CMOS photodiodes, even though the software is more complex to fully setup.

Taken together, these results indicate that the computational advantage of the simplified approaches is accompanied by a relatively small deviation from the full-wave reference for the planar CMOS stacks investigated. The results therefore provide a quantitative basis for assessing the use of simplified models during optical detection, rather than relying only on visual agreement between the simulated spectra. For the structures investigated in this work, the differences in the predicted optical transmittance observed between the three numerical approaches are negligible from a practical design perspective, making computational efficiency the primary condition for method selection.

## 4. Conclusions

This work presented a comparative analysis of three numerical approaches, Wave Optics, Ray Optics, and TFCalc (TMM), for modelling optical transmission through complex multilayer dielectric stacks, representative of TSMC 65 nm and TSMC 180 nm CMOS technologies. Rather than introducing a new numerical formulation, the study evaluated the extent to which established approaches can reproduce the optical response of realistic CMOS dielectric stacks and assessed their suitability for different modelling requitements. The comparison considered spectral transmittance, manufacturing thickness tolerances, angular illumination effects, computational efficiency and model agreement under identical simulation conditions.

Despite relying on fundamentally different physical formulations, all three approaches produced highly consistent transmittance predictions for the planar multilayer structures investigated. Process-induced thickness variations primarily shifted the spectral position of the interference peaks, while preserving the overall interference behavior. The angular illumination response analysis further demonstrated that the transmittance strongly depends on the light angle of incidence, reflecting the continuous evolution of the interference conditions within the multilayer dielectric stack. However, when uncollimated practical LED illumination was considered through angular averaging, these oscillatory features were substantially smoothed, indicating that experimental responsivity measurements under such illumination are expected to reveal a much more uniform spectral response than predicted under ideal normal incidence conditions. This smoothing should not be expected for collimated laser illumination, for which the incident angular distribution is narrow, and the interference features can remain pronounced. Together, these results demonstrate that both fabrication tolerance and realistic illumination conditions should be considered when accurately predicting the optical performance of CMOS photodiodes.

From a practical design perspective, the differences in the predicted transmittance between the three numerical approaches were negligible for the planar multilayer dielectric stacks investigated. Consequently, the choice of modelling strategy should primarily depend on the complexity of the optical structure and the available computational resources rather than on predictive accuracy alone. While Wave Optics remains the preferred solution for arbitrary non-planar or three-dimensional geometries, the TMM implemented in TFCalc provides the most favorable balance between accuracy and computational efficiency for standard planar CMOS dielectric stacks. These findings establish practical guidelines for selecting the most appropriate numerical modelling strategy during the design and optimization of CMOS photodiodes, enabling accurate optical performance prediction while substantially reducing computational time.

Despite the agreement observed between the numerical approaches, two aspects remain to be addressed in future work. First, experimental measurements on fabricated silicon photodiodes would provide an independent comparison with the predicted spectral transmission and help assess the extent to which the simulated stack transmission is reflected in the measured photodiode responsivity, while also accounting for uncertainties in the optical properties and fabricated layer thicknesses. Second, the present analysis is restricted to laterally uniform planar dielectric stacks and does not account for structures surrounding the active area of practical CMOS photodiodes, such as metal interconnects and other layout-dependent features. Future work will therefore consider representative photodiode structures including such surrounding metal elements, using Wave Optics to investigate their influence on the optical transmission.

## Figures and Tables

**Table 1:** Optical properties of the five dielectric materials comprising the TSMC 65 nm technology stack [15].

Id.	Dielectric Constant	Refractive Index
D1	2.9	1.70
D2	4.2	2.05
D3	4.5	2.12
D4	5.0	2.24
D5	8.1	2.85

**Table 2:** Optical properties of the four dielectric materials comprising the TSMC 180 nm technology stack [14].

Id.	Dielectric Constant	Refractive Index
D1	3.7	1.92
D2	4.0	2.00
D3	4.2	2.05
D4	7.9	2.81

**Table 3:** Number of elements and quality statistics of the mesh for the two CMOS technologies with the average thickness of each layer for the Wave Optics model.

CMOS Technology	Number of Elements	Average Element Quality
TSMC 65 nm	64779	0.9438
TSMC 180 nm	60839	0.9558

**Table 4:** Comparison of computational times required to execute the standard transmittance study across the three numerical approaches and accuracy metrics against the reference Wave Optics model.

Numerical Model	Computational Time	MAD (%)	MRD (%)
Wave Optics	~3 h and 53 min	Reference	Reference
Ray Optics	~40 s	0.49	1.19
TFCalc	~1 s	2.25	5.55

## Data Availability

The data supporting the study findings can be provided upon request to the corresponding author.

## Supplementary Materials

The following supporting information can be downloaded at: https://www.mdpi.com/article/10.3390/s26206591/s1, Figure S1: Spectral transmittance of the TSMC 65 nm technology at the (**a**) minimum and (**b**) maximum manufacturing thickness limits, comparing the three numerical models; Figure S2: Spectral transmittance of the TSMC 180 nm technology at the (**a**) minimum and (**b**) maximum manufacturing thickness limits, comparing the three numerical models.

## Abbreviations

The following abbreviations are used in this manuscript:

CMOS	Complementary Metal Oxide Semiconductor
FDTD	Finite-Difference Time-Domain
FEM	Finite Element Method
IMD	Inter-Metal Dielectric
LED	Light-Emitting Diode
MAD	Mean Absolute Difference
MRD	Mean Relative Difference
MUMPS	MUltifrontal Massively Parallel Sparse
NIR	Near-InfraRed
RCWA	Rigorous Coupled-Wave Analysis
TE	Transverse Electric
TM	Transverse Magnetic
TMM	Transfer-Matrix Method
TSMC	Taiwan Semiconductor Manufacturing Company
