# Supplementary material for: Numerical Characterization of Optical Transmittance Through Planar Multilayer Dielectric Stacks on CMOS Photodiodes
Source: Sensors (Basel). 2026 Oct 17;26(20):6591. doi: 10.3390/s26206591 (PMC13708913; doi:10.3390/s26206591)
Supplement: Supplementary file 1 [file sensors-26-06591-s001.zip › sensors-4615874-supplementary.pdf]

Numerical Characterization of Optical Transmittance through Planar Multilayer Dielectric Stacks on CMOS Photodiodes

João Fernandes [1], Gabriel M. Ferreira [1,2], J. P. Carmo [3], Graça Minas [1,4,*] and Susana Catarino [1,4]

1 Center for Microelectromechanical Systems (CMEMS), School of Engineering, Campus de Azurém, University of Minho, Guimarães 4800-058, Portugal
2 International Iberian Nanotechnology Laboratory, INL, Braga 4715-330, Portugal
3 Group of Metamaterials Microwaves and Optics (GMeta), SEL, Univ. São Paulo, Av. Trabalhador S.-Carlense, Nr.400, CEP13566-590, São Carlos-SP, Brazil
4 LABBELS—Associate Laboratory, Braga 4800-122, Portugal
* Correspondence: gminas@dei.uminho.pt

Supplementary Material

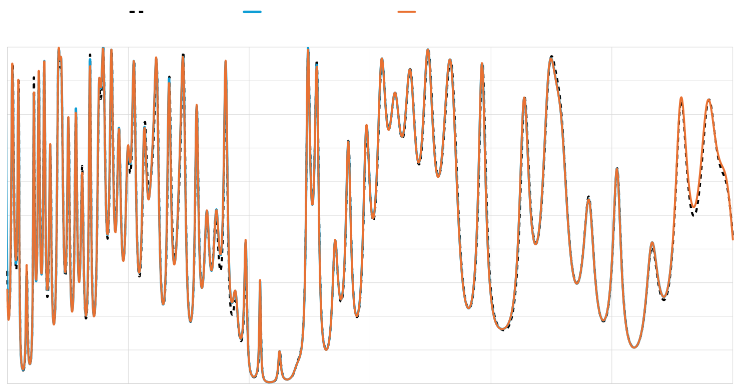

(a)

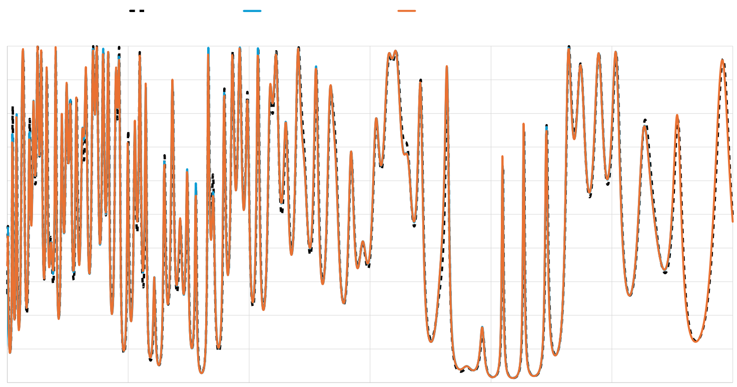

(b)

Figure S1. Spectral transmittance of the TSMC 65 nm technology at the **(a)** minimum and **(b)** maximum manufacturing thickness limits, comparing the three numerical models.

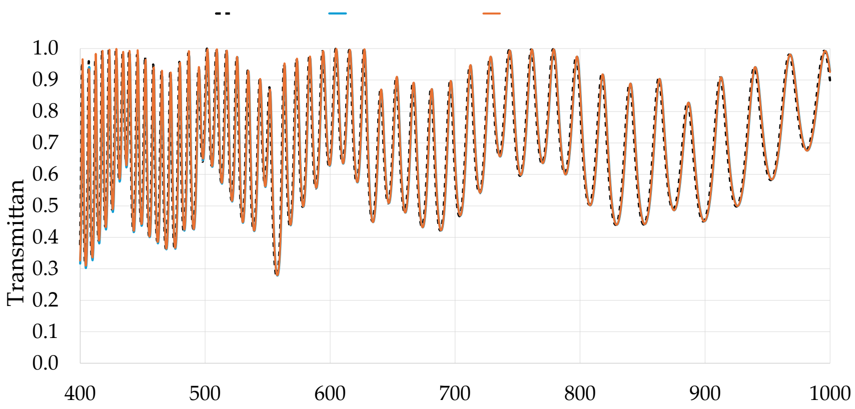

(**a**)

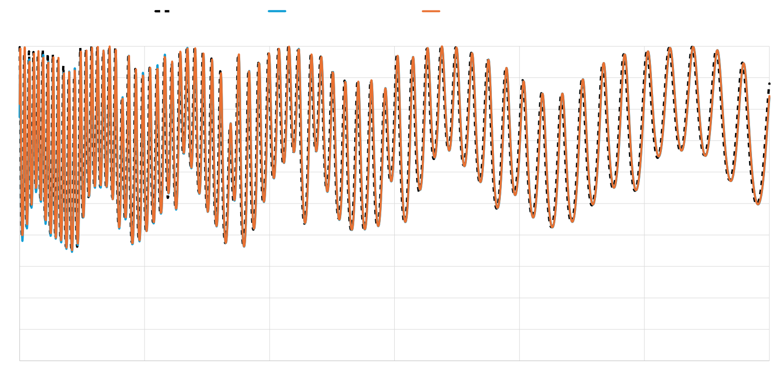

(**b**)

Figure S2. Spectral transmittance of the TSMC 180 nm technology at the **(a)** minimum and **(b)** maximum manufacturing thickness limits, comparing the three numerical models.
